# Role of intestinal microecology in the regulation of energy metabolism by dietary polyphenols and their metabolites

**DOI:** 10.29219/fnr.v63.1518

**Published:** 2019-02-14

**Authors:** Shaoling Lin, Zhengyu Wang, Ka-Lung Lam, Shaoxiao Zeng, Bee K. Tan, Jiamiao Hu

**Affiliations:** 1 College of Food Science, Fujian Agriculture and Forestry University, Fuzhou, China; 2 School of Life Sciences, The Chinese University of Hong Kong, Shatin, Hong Kong SAR, China; 3 Departments of Cardiovascular Sciences, Health Sciences and Leicester Diabetes Centre, College of Life Sciences, University of Leicester, University Road, Leicester, United Kingdom

**Keywords:** polyphenols, gut microecology, energy metabolism

## Abstract

**Background:**

Polyphenols are a class of plant secondary metabolites with a variety of physiological functions. Polyphenols and their intestinal metabolites could greatly affect host energy metabolism via multiple mechanisms.

**Objective:**

The objective of this review was to elaborate the role of intestinal microecology in the regulatory effects of dietary polyphenols and their metabolites on energy metabolism.

**Methods:**

In this review, we illustrated the potential mechanisms of energy metabolism regulated by the crosstalk between polyphenols and intestinal microecology including intestinal microbiota, intestinal epithelial cells, and mucosal immune system.

**Results:**

Polyphenols can selectively regulate the growth of susceptible microorganisms (eg. reducing the ratio of Firmicutes to Bacteroides, promoting the growth of beneficial bacteria and inhibiting pathogenic bacteria) as well as alter bacterial enzyme activity. Moreover, polyphenols can influence the absorption and secretion of intestinal epithelial cells, and alter the intestinal mucosal immune system.

**Conclusion:**

The intestinal microecology play a crucial role for the regulation of energy metabolism by dietary polyphenols.

## Popular scientific summary

- Dietary polyphenols have an important impact on energy metabolism.
- Dietary polyphenols may affect host energy metabolism via regulating intestinal microecology.
- Crosstalk between polyphenols and gut microbiota may have regulatory effects on host metabolic control.

## 

Polyphenols are plant secondary metabolites that widely exist in vegetables and fruits with potential contribution to the prevention of chronic diseases, including cardiovascular disease, cancer, obesity, and diabetes (1, 2). A number of polyphenols are minimally absorbed, and the rest are transformed by intestinal bacteria into other bioactive polyphenol metabolites. These polyphenols and their metabolites can influence the type and quantity of intestinal microbial species which in return may affect their bioavailability and bioactivity.

Recent findings also suggest the relationship between polyphenols and the intestinal flora in the development of obesity and obesity-related metabolic diseases. Intestinal bacterial modulation was shown to trigger obesity in both humans and animals (3, 4), and higher ratio of *Firmicutes* and *Bacteroides* phyla was found to be correlated with increased body weight (5, 6). Recent studies also revealed the selective growth-stimulating effect of gut microbes by polyphenols, leading to obesity prevention (7, 8). Therefore, polyphenols, a potential ‘metabolic prebiotics’, could provide beneficial effects to hosts (such as weight loss) by reshaping the gut microbial communities (9). In this review, we summarized recent studies investigating the effects of dietary polyphenols and their metabolites to gut microecology and energy metabolism.

## Intestinal microecology and energy metabolism

The ‘intestinal microecology’ consists of three parts: intestinal microbiota, intestinal epithelial cells, and mucosal immune system that together form the intestinal mucosal barrier (10). The intestinal flora may serve the most important roles in intestinal microecology. At least 500–1,000 different bacterial species have been identified to be present in the human gastrointestinal tract, and up to 98% of intestinal flora can be classified into four phyla: *Firmicutes* (64%), *Bacteroidetes* (23%), *Proteobacteria* (8%), or *Actinobacteria* (3%) (11–13). Intestinal dysbiosis is considered as an important factor inducing metabolic diseases including obesity, chronic inflammation and insulin resistance, secondary to dietary changes (14–16). On the other hand, the roles of intestinal epithelial cells in the intestinal microecology cannot be overlooked. For example, secretory mucin, lysozyme, and defensins could inhibit the growth of certain intestinal microbes and prevent their intestinal adhesion; meanwhile, these secreted protein/peptides are also associated with the release of interleukin factors including IL-1α, IL-1β, IL-6, IL-8, and IL-10, which are all involved in host inflammatory response, adipose tissue energy metabolic disorder and development of insulin resistance (10). Finally, the intestinal mucosal immune system, one of the major immune organs, functions to exclude and provide tolerance to antigens (17). It has been reported that long-term intake of high-fat diets will increase the permeability of the intestinal mucosa, resulting in endotoxemia, causing chronic inflammation, and eventually inducing metabolic disorders including obesity and insulin resistance (18). The increase of mucosal permeability was also found to be positively correlated with the degree of steatosis and fat accumulation in the liver (19). Taken together, the intestinal microecology plays multiple and yet important roles in the regulation of energy metabolism.

## The absorption and metabolism pathway of polyphenols in the intestine

**Fig. 1:**
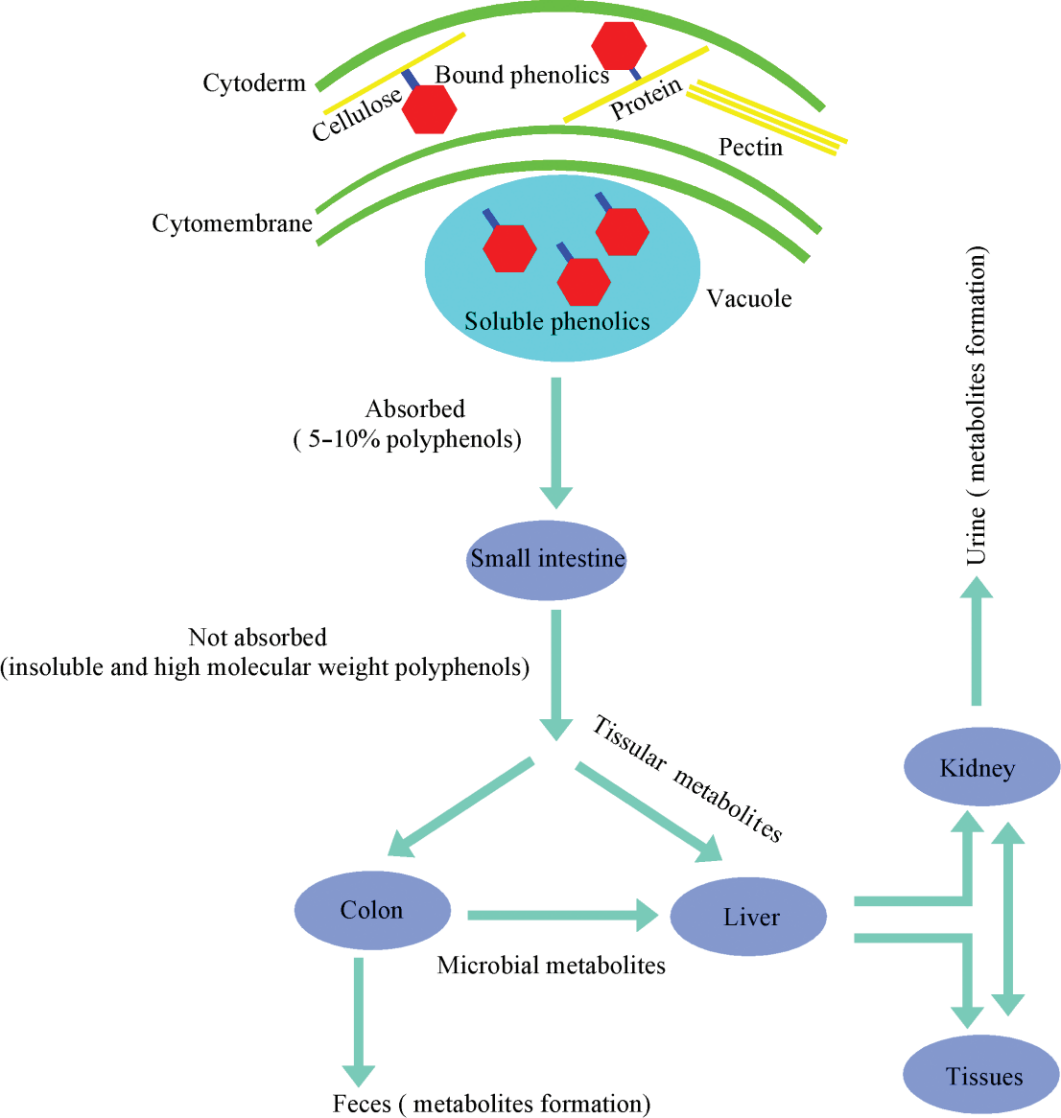
The metabolic pathway of dietary polyphenols in humans. A small portion of polyphenols are directly absorbed by the small intestine. The majority of polyphenols (the insoluble and high molecular weight polyphenols) undergo extensive metabolism by gut microflora or tissues before being excreted, which represents at least 90–95% of the polyphenol intake.

**Fig. 2:**
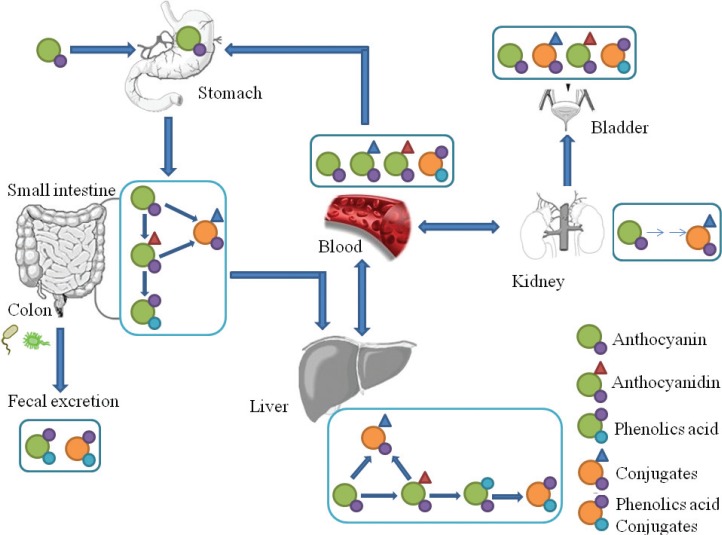
The hypothetic pathways of anthocyanin absorption and metabolism based on literature review (40, 41). Anthocyanin undergoes extensive metabolism in the body; the stomach exhibited only native anthocyanin, while in other organs native anthocyanin and its metabolites (phenolic acid or conjugates) were detected before being excreted.

Plant-based foods contain polyphenols in both soluble and insoluble-bound forms. As shown in Fig. 1, soluble polyphenols are mainly found in the vacuole. Dietary intake of free and soluble polyphenols can be rapidly absorbed by active transport or passive diffusion and distributed throughout the body, bringing health benefits such as oxidative inhibition of low-density lipoprotein (LDL), cholesterol and liposomes (20, 21). In contrast, insoluble polyphenols are structurally bound with proteins, cellulose, pectin, and other macromolecules in the cell wall *via* ether, ester or C-C bonds and released as phenolic glycosides by colonic microflora or enzymes to exert their health benefits (22–24). In fact, insoluble and high molecular weight polyphenols, which account for approximately 90–95% of the total polyphenols intake, are metabolized by gut microflora rather than being absorbed by the gastrointestinal tract (25, 26). As a consequence, a myriad of diverse groups of dietary polyphenol-derived metabolites are found in human and animal excrement (feces or urine), as shown in Table 1. Taking anthocyanin as an example, it undergoes extensive metabolism in the body before being excreted; the proportion of intact anthocyanin excreted in urine was estimated to be lower than 0.1% of the intake (Fig. 2).

**Table 1:** Metabolites of phenolics compounds *via* gut microbiota *in vivo* or *in vitro*

Polyphenols	Type of Study	Metabolites	References
Baicalin	*In vitro* study (humans feces)	Baicalein	(27)
Epicatechin	*In vitro* study (humans feces)	(-)-5-(3’,4’-dihydroxyphenyl)-γ-valerolactone,5-(3,4-dihydroxyphenyl)-γ-valeric acid,3-(3-hydroxyphenyl)propionic acid,4-hydroxyphenylacetic acid	(28)
Apigenin	Animal study (urine)	P-hydroxyphenylacetic acid, P-hydroxycinnamic acid,P-hydroxybenzoic acid	(29)
Quercetin	Animal study (urine)	4-ethylphenol, Benzoic acid,4-ethylbenzoic acid	(30)
Catechin	Human intervention (urine)	(-)-5-(3′,4′,5′-trihydroxyphenyl)-γ-valerolactone(M4),(-)-5-(3′,4′-dihydroxyphenyl)-γ-valerolactone	(31)
Naringenin	*In vitro* study (rat feces)	Phenylacetic acid, P-hydroxyphenylacetic acid, Protocatechuic acid	(32)
Naringin	*In vitro* study (humans feces)	3-(4-hydroxyphenyl)-propionic acid,3-phenylpropionic acid	(33)
Rutin	*In vitro* study (humans feces)	3-(3-hydroxyphenyl)-propionic acid,3-hydroxyphenylacetic acid	(33)
Rutin	*Escherichia coli*	3,4-dihydroxyphenylacetic acid	(34)
Daidzein	*In vitro* study (rat feces)	Dihydrodaidzein	(35)
Anthocyanin	*In vitro* study (humans feces)	Gallic, syringic and p-coumaric acids.	(36)
Chlorogenic acid	*In vitro* study (humans feces)	3-(3-hydroxyphenyl)-propionic acid	(33)
Caffeic acid	*In vitro* study (humans feces)	Hydroxyphenylpropionic and Benzoicacids	(37)
Ferulaic acid	*Lactobacillus* and *Bifidobacterium*	Coumaric acid and Caffeic acid	(38)
Ellagic acid	*In vitro* study (humans feces)	Urolithin(A)	(39)

## Energy metabolism regulatory mechanisms involving dietary polyphenols and intestinal bacteria

### Polyphenols reshape the composition and diversity of gut bacterial communities

The metabolism of polyphenols by gut bacteria involves hydrolysis of glycosidic bonds and decomposition of polyphenol heterocycle (42). Glycans, the products of glycoside cleavage, are essential nutrients for most intestinal microbes (43). Evidence suggests that dietary polyphenols play a crucial role in modulating the gut microbial community such as alleviating pathogen growth, regulating commensal bacteria and probiotics, and enhancing host-microbial interactions, ultimately leading to beneficial effects such as weight loss (9, 44).

**Fig. 3:**
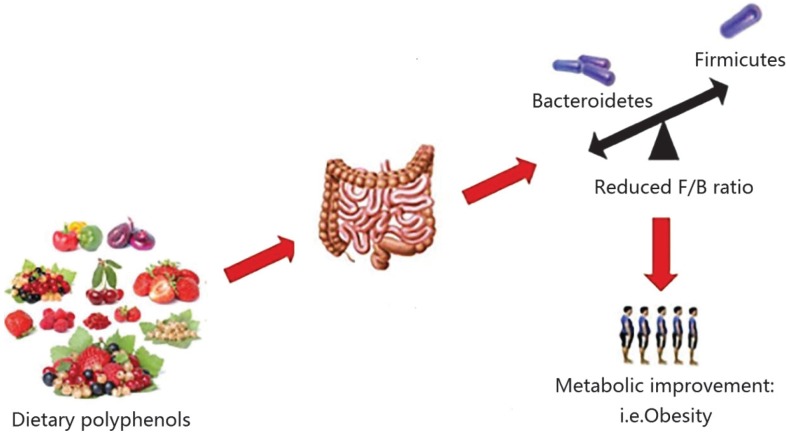
Dietary polyphenols inhibit the metabolic disease related to obesity by regulating the intestinal microflora ecology, for example, lowering *Firmicutes*/*Bacteroidetes* ratio (49, 50).

The gut microbiota is dominated by anaerobic bacteria, mainly the *Firmicutes* and *Bacteroidetes* phyla. A reduced *Firmicutes*-to-*Bacteroidetes* ratio has been associated with improved glucose levels, alleviated fat accumulation and decreased body weight (45–48). The intake of fruits and vegetables, such as apples, pears, strawberry, grapefruit, eggplant, green pepper, all of which are rich in polyphenols, may promote weight loss in obese patients. These effects were possibly attributed to the ratio of *Firmicutes/Bacteroidetes* lowering caused by the regulation of phenolic compounds (Fig. 3). A human intervention study performed with the administration of de-alcoholized red wine, an excellent source of anthocyanins, revealed a significant lowering of blood pressure, serum triglycerides (TG), and high-density lipoprotein (HDL) cholesterol level, which may be partly due to the greater reduction in *Firmicutes* than *Bacteroidetes* (51). It has also been found that quercetin administration led to a reduction in the *Firmicutes/Bacteroidetes* ratio and this was associated with reduced body weight gain and serum insulin levels in patients who consumed high-fat and high-sucrose diets (52). Similarly, Zhao et al. (53) found that the combined actions of the polyphenols quercetin and resveratrol lowered the *Firmicutes/Bacteroidetes* ratio in rats fed with high-fat diets, thereby decreasing their subsequent weight gain. Thus, diets containing different polyphenols might reshape the gut microbiota in various ways; however, the reduction in the ratio of *Firmicutes* to *Bacteroidetes* resulting from polyphenol administration might contribute to weight loss in obese individuals and aid in maintaining a normal body weight.

Probiotics intake is also found to be correlated with weight loss (54–58). Polyphenols are known to alter a number of *Bifidobacterium* and *Lactobacillus* in the intestinal tract. For example, flavanols promoted the growth of *Lactobacillus spp.* and *Bifidobacterium spp.*, which may partly be responsible for the observed reductions in plasma concentrations of C-reactive protein (CRP) (59); CRP is produced by adipose tissue and largely occurs under the transcriptional control of interleukin-6 (60, 61). In addition, these changes in *Bifidobacterium* and *Lactobacillus* abundance are also associated with significant reduction in plasma triacylglycerol level, which may contribute to the benefits associated with dietary polyphenols (62–64). In addition, polyphenol-rich pomegranate peel extract was found to increase the caecal pool of *Bifidobacteria* accompanied with reduced serum level of total cholesterol (TC) and LDL cholesterol induced by high-fat diet in mice (65). Another research group observed a significant increase in the proportion of *Bifidobacterium* in obese patients after consumption of red wine polyphenol for 4 weeks, and reported that *Bifidobacterium* positively correlated with HDL cholesterol levels (51). Thus, polyphenols have the ability to promote the growth of probiotic bacteria, contribute to the improvement of intestinal barrier function and prevent or treat metabolic syndrome and obesity.

*Enterobacter* genus (*proteobacteria* phylum), presented at higher baseline level in obese compared to healthy subjects, has been found to induce obesity and metabolic syndrome in human hosts (66). For example, *Enterobacter cloacae* produces endotoxins, causing non-obese aseptic mice to develop severe obesity, inducing inflammation and insulin resistance in mice, downregulating genes involved in fat catabolism, and activating lipogenesis genes (67, 68). Till now, available experimental evidence showed that polyphenols have an inverse relationship with intestinal *Enterobacter*. The promotion of *Enterobacter* was strongly inhibited by the presence of tea phenolics (epicatechin, catechin, 3-O-Me gallic acid, gallic acid, caffeic acid, and so on) as well as their aromatic metabolites including 3-(4-OH phenyl) propionic acid, 3-Phenylpropionic acid, and 4-OH phenylacetic acid (69). Moreover, consumption of polyphenol-containing red wine or de-alcoholized red wine normalized the *Enterobacter* and improved blood pressure and blood glucose dysregulation in patients with the metabolic syndromes (70). Furthermore, a combination of quercetin and resveratrol can cease the relative population increase of *Enterobacter cloacae* induced by high-fat diet, and this may relate to the lowering of body weights, serum lipids, and inflammatory cytokines levels (53). Thus, lowering of the relative *Enterobacte*r population in the human intestinal tract may serve as another mechanism for polyphenols to rectify metabolic abnormalities. *Enterobacter* could be the new target for the prevention and treatment of obesity and related diseases.

*Akkermansia muciniphila* (*A. muciniphila*), a member of the *Verrucomicrobia* phylum, is believed to have anti-inflammatory and anti-obesity effects in humans and rodents (71, 72). The proportion of *A. muciniphila* is found to be around 3–5% in human digestive tract, but significantly reduced in obese individuals (73, 74). *A. muciniphila* could increase the thickness of intestinal walls by stimulating the secretion of mucin, which hinders food absorption (74). A lower relative abundance of *A. muciniphila* tends to induce poor performance in obesity-associated metabolic phenotypes such as insulin resistance, inflammation, and ponderal growth (75–77). As an obligate anaerobe, *A. muciniphila* indeed is susceptible to the presence of free oxygen radicals. Intriguingly, unabsorbed polyphenols could scavenge reactive oxygen species, thereby allowing *A. muciniphila* to thrive (78, 79). Some recent literature further supported this hypothesis; an *in vivo* study showed ellagic acid (a metabolite of pomegranate ellagitannins) promoted the growth of *A. muciniphila* (80). Polyphenol-rich cranberry extract was also found to improve insulin tolerance and attenuate intestinal inflammation in mice fed with high-fat/high-sucrose diet, and these effects are linked to the expansion of *Akkermansia* population (81). Consequently, dietary polyphenols very likely play an important role in modulating the relative abundance of *A. muciniphila* and therefore the control of host energy metabolism. Nevertheless, this link between changes in *A. muciniphila* population and weight loss awaits further experimental confirmation.

### Polyphenols are metabolized to generate short-chain fatty acids via intestinal microbiota

The short-chain fatty acids (SCFAs) are beneficial for the prevention of obesity-related metabolic diseases (82, 83). Acetic acid is the major product of intestinal saccharolytic fermentation, which reduces appetite, and can be absorbed and utilized by peripheral tissues in the host (84). Propionic acid, one of the major fermentation products by *Bacteroides*, is further decomposed and metabolized in the liver after being absorbed into the blood, regulating the conversion of pyruvate to glucose and potentially inhibiting fat synthesis (85). Butyric acid, one of the major fermentation products by *Firmicutes*, is the primary energy source of colon epithelial cells (86). It is worth emphasizing that a large proportion of dietary polyphenols may be metabolized in the colon, and broken down into small molecules, including organic acids such as lactate, succinate, pyruvate, butyrate, fumarate, and acetate (87, 88). According to Bleut et al. (89), anaerobic bacteria in gut can cleave the ring structure of several flavonoids into hydroxyphenylacetic and hydroxyphenylpropionic acids, as well as into acetate and butyrate. Coincidentally, supplementation of quercetin and fructooligosaccharides enhanced the production of SCFAs, especially butyric acid, whereas supplementation of catechin and fructooligosaccharides significantly increased the production of propionic acid compared to administration of fructooligosaccharides alone (90). Besides, anthocyanins, a compound being used prophylactically and therapeutically, is also found to exhibit positive effects on the production of SCFAs, including acetic, propionic, and butyric acids, by regulating the intestinal microbial flora (82). In addition, polyphenols can induce changes in gut microbiota and therefore the production of SCFAs, leading to an upregulation of phosphorylated AMP-activated protein kinase (91, 92), which takes an up-stream and yet strong part in the energy metabolic pathways.

### Polyphenols influence the activity of intestinal microbial enzymes

Intestinal microbiota affects the host physiological processes *via* a wide range of secretory enzymes, including hydrolase, oxidoreductase, lyase and transfer enzymes to regulate host energy metabolism (77, 93). Intriguingly, dietary polyphenols also have significant effects on the intestinal microbial enzymes. For example, polyphenols can inhibit bacterial enzyme activity by metal ions (iron and cobalt) chelation, leading to altered microbial metabolism (94, 95). The catechin epigallocatechin gallate exhibits strong antibiotic activity against *Stenotrophomonas maltophilia* (a kind of bacteria linked to inflammation) *via* inhibition of its dihydrofolate reductase (96, 97). Also, the increased abundance of *Bacteroidetes* by polyphenols may contribute to energy homeostasis due to the large number of glycan-degrading enzymes such as glycoside hydrolases and polysaccharide lyases possessed by *Bacteroidetes* (43). Thus, this might be another mechanism by which polyphenols exert weight-reducing effect *via* increasing *Bacteroidetes* abundance in gut (50).

### Polyphenols influence fasting-induced adipose factor via intestinal microbiota

**Fig. 4:**
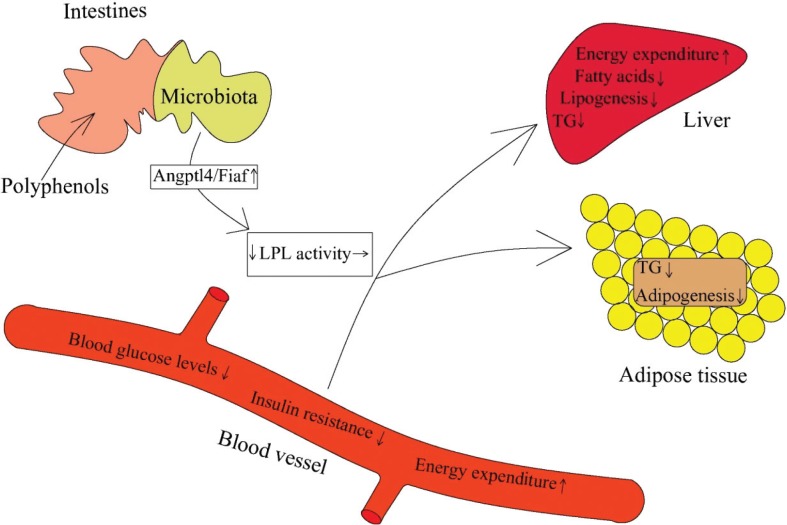
Polyphenols change *fiaf* gene expression *via* reshaping microbiota structure (87, 88, 98–100). The active *fiaf*, an inhibitor of LPL, can promote lipid clearance in blood, suppress hepatic lipogenesis and contribute to the release of fatty acids and triacylglycerol from circulating lipoproteins in adipose tissue.

Fasting-induced adipose factor (*Fiaf*), also known as angiopoietin-like protein 4 (Angptl4), inhibits adipocytokine lipoprotein lipase activity and promotes fatty acid oxidation. Polyhenols and their metabolites can alter intestinal *Fiaf* expression by affecting the diversity of gut microbiota, which then can lead to the changes in lipoprotein lipase activity in gut and modulate host energy metabolism (Fig. 4). Recent evidence indicated that adding quercetin to a high-fat, high-sucrose diet significantly increased the expression of *Fiaf*, which was associated with beneficial changes in the gut microbiota (101); the microbial populations of *Bifidobacterium* and *A. muciniphila* were credited with the increase of intestinal *Fiaf* expression by secreting bioactive compounds (102, 103). For example, anthocyanin was shown to activate *Fiaf* expression in gut epithelium by means of increasing the growth of *Bifidobacterium*, and reducing fat storage (104). Furthermore, research indicated that resveratrol has the potential to attenuate mRNA expression of fatty acid synthesis genes and switch on to lipolysis-related genes in the host, which may be driven by increased *Fiaf* expression in the intestine (105). These changes in gene expression may be responsible for the prebiotic effect of resveratrol on the gut microbiota. In addition, the microbial metabolites of polyphenols, such as propionate and butyrate, can also promote the expression of the *Fiaf* in gut epithelial cell lines (106). Therefore, polyphenols and their metabolites may be able to influence intestinal *Fiaf* expression, and through this mechanism, regulate energy metabolism.

### Energy metabolism regulatory mechanisms involving dietary polyphenols and intestinal mucosal epithelial cell

### Polyphenols modulate glucagon-like peptide-1 secretion

Glucagon-like peptide-1 (GLP-1) is an endogenous insulinotropic peptide secreted from the intestine L cells in response to food intake (107). GLP-1 analogues promote insulin secretion and decrease glucagon secretion in a glucose-dependent manner (108). Current findings also demonstrated that resveratrol given orally exerts an anti-diabetic effect linked to increased intestinal levels of GLP-1 (109). Furthermore, anthocyanin is propitious to energy homeostasis, possibly by inducing the secretion of GLP-1 (110). In addition, polyphenol metabolites such as SCFAs in the intestine were also found to stimulate the release of GLP-1, which further inhibits appetite and food intake, delays gastric emptying and increases the sensation of fullness (87, 88, 111, 112). Therefore, the beneficial effects of polyphenols may be on the grounds of a GLP-1 receptor-dependent manner.

### Polyphenols modulate sodium-coupled glucose transporter 1 expression

Sodium-coupled Glucose Transporter 1 (SGLT1), a major Na-dependent glucose co-transporter on the brush border of intestinal epithelial cells, regulates intestinal glucose uptake and glucose-dependent incretin secretion like GIP-1 (113). Polyphenols might play a significant role in controlling the dietary glucose uptake in the intestinal tract by attenuating SGLT1 expression (114, 115). Coincidentally, flavonoids, with well-documented anti-diabetic activities, can hamper glucose uptake mediated by the intestinal glucose transporter SGLT1 in mouse (116). Also, tea polyphenols have been shown to inhibit the glucose transport activity of SGLT1, with the most pronounced inhibition by epicatechin gallate (117). In addition, polyphenols include phlorizin, quercetin, kaempferol, phloretin, and cholorgenic acid, which are found to inhibit SGLT1 expression and diminish glucose responses in mice and humans (118). Streptozotocin-induced diabetic mice are given a diet containing 0.5% phloridzin for up to 14 days; blood glucose levels were significantly improved, probably through the decreased expression of SGLT1 in the small intestine (119). Based on these findings, polyphenols may act as potent inhibitors of glucose absorption by suppressing the SGLT1 sugar transporters, and serve as a promising treatment option to obesity and metabolic diseases (120, 121).

### Polyphenols modulate fructose/glucose transporter expression

Experimental evidence suggested consumption of high-fructose products in rat models or in humans could lead to the development of metabolic syndrome, which is characterized by obesity, high blood pressure, and increased serum glucose, insulin and TG levels (122). Two glucose/fructose transporters (GLUT2, which transports both glucose and fructose, and GLUT5, which transports fructose only) mediate intestinal glucose/fructose transport from the intestinal lumen into enterocytes. Flavanols are the potent non-competitive inhibitors of the intestinal sugar transporters (116). Some recent evidence indicated that quercetin, apigenin, chrysin, curcumin, and bisdemethoxy can reduce the expression of GLUT2 or GLUT5 genes to interfere with fructose absorption *via* the intestinal–epithelial cells (123, 124). In addition, some polyphenol-rich foods were shown to improve glucose control by efficiently attenuating glucose transport across intestinal cells through interaction with the GLUT transporter family (125). *In vitro* studies also showed that the polyphenols contained in blackcurrant and apple extracts inhibited GLUT-mediated glucose uptake in Caco-2/TC7 cells (a frequently used cellular model of the small intestine), and cinnamon polyphenol extract can affect immune responses by regulating GLUT gene expression (126, 127). Therefore, dietary polyphenols may be beneficial to the host by regulating the rate of intestinal sugar absorption and preventing excessive glucose/fructose uptake; this may lead to reducing the risk of obesity, diabetes and the metabolic syndrome.

### Energy metabolism regulatory mechanisms involving dietary polyphenols and intestinal mucosal immune system

Latest studies have correlated the impairments in intestinal immune homeostasis and the mucosal barrier with increased activation of inflammatory pathways and the pathogenesis of insulin resistance (128). Studies conducted in *in vivo* and *in vitro* models have provided evidence that polyphenol as well as polyphenol-rich foods have beneficial effects on gut health, such as modulation of mucosal immune and inflammatory response *via* downregulation of inflammatory cytokines and suppression of pro-inflammatory signaling pathways (129–131). Lipopolysaccharide (LPS), an endotoxin released by gram-negative bacteria, is important for the induction of gut mucosal permeability by provoking inflammatory responses and aggravating inflammation-related chronic conditions such as obesity and insulin resistance (132, 133). It has been proved that polyphenols might ameliorate the development of metabolic endotoxemia by interfering with LPS in the gut lumen (134). For instance, the supplementation of anthocyanin-rich fruit can alleviate low-grade inflammation by upregulating the interleukin-10 gene expression and downregulating inflammatory markers (interleukin-6, tumor necrosis factor-α) in the colon with an increased growth of *Lactobacillus spp* in the offspring (135). In addition, polyphenol metabolites showed a strong inhibition toward LPS activation. Ferulaldehyde, a water-soluble degradation product of polyphenols, inhibited the LPS-induced inflammatory response in mice (136). Urolithins, another group of gut microbiota-derived metabolites of ellagitannins, are responsible for anti-inflammatory properties (137). Also, the 3-O-methylquercetin, a metabolite of quercetin, showed stronger potential in inhibiting LPS-mediated activation of macrophage U937 cells compared to quercetin itself (138). Therefore, dietary polyphenols as well as their metabolites may act a key role in the intestinal mucosal immune system.

## Conclusions

Current evidence has strongly supported the correlation of the occurrence of obesity with a shift in intestinal microecology. Polyphenols and their diverse metabolites have profound influence on the diversity and complexity of the intestinal microflora. Various studies have been carried out to understand the response of the gut microbiota with polyphenol administration as well as to identify the key microorganisms involved. It is clear that dietary polyphenols and their metabolites contribute to the maintenance of energy homeostasis and gut health through modulation of the gut microbiome, intestinal epithelial cellular function, and the mucosal immune system. Although the detailed mechanism by which polyphenols interact with the gut microecology is still not yet well characterized, polyphenols appear to influence energy metabolism and promote weight loss by re-structuring the intestinal microecology. This may provide a new viewpoint for obesity treatment *via* polyphenol interventions.
